# Identifying mutant-specific multi-drug combinations using comparative network reconstruction

**DOI:** 10.1016/j.isci.2022.104760

**Published:** 2022-07-15

**Authors:** Evert Bosdriesz, João M. Fernandes Neto, Anja Sieber, René Bernards, Nils Blüthgen, Lodewyk F.A. Wessels

**Affiliations:** 1 Bioinformatics, Computer Science, VU Amsterdam, De Boelelaan 1111, Amsterdam 1081 HV, the Netherlands; 2 Division of Molecular Carcinogenesis, The Oncode Institute, The Netherlands Cancer Institute, Plesmanlaan 121, Amsterdam 1066 CX, the Netherlands; 3 Institute of Pathology, Charite Universitatsmedizin, Chariteplatz 1, Berlin 10117, Germany; 4 IRI Life Sciences, Humboldt University of Berlin, Philippstraße 13, Berlin 10115, Germany; 5 Berlin Institute of Health, Anna-Louisa-Karsch-Straße 2, Berlin 10178, Germany; 6 Faculty of EEMCS, Delft University of Technology, Mekelweg 4, Delft 2628 CD, the Netherlands

**Keywords:** Bioinformatics, Pharmacoinformatics, Systems biology, In silico biology

## Abstract

Targeted inhibition of aberrant signaling is an important treatment strategy in cancer, but responses are often short-lived. Multi-drug combinations have the potential to mitigate this, but to avoid toxicity such combinations must be selective and given at low dosages. Here, we present a pipeline to identify promising multi-drug combinations. We perturbed an isogenic PI3K mutant and wild-type cell line pair with a limited set of drugs and recorded their signaling state and cell viability. We then reconstructed their signaling networks and mapped the signaling response to changes in cell viability. The resulting models, which allowed us to predict the effect of unseen combinations, indicated that no combination selectively reduces the viability of the PI3K mutant cells. However, we were able to validate 25 of the 30 combinations that we predicted to be anti-selective. Our pipeline enables efficient prioritization of multi-drug combinations from the enormous search space of possible combinations.

## Highlights

• Systematics drug perturbations measurements in isogenic PI3K wild-type and mutant cells
• Mutant-specific reconstruction and quantification of signal transduction networks
• Short-term signaling response is predictive for longer term cell viability
• 25 out of 30 model predictions of mutant-specific multi-drug combinations validated

## Graphical abstract

**Figure:**
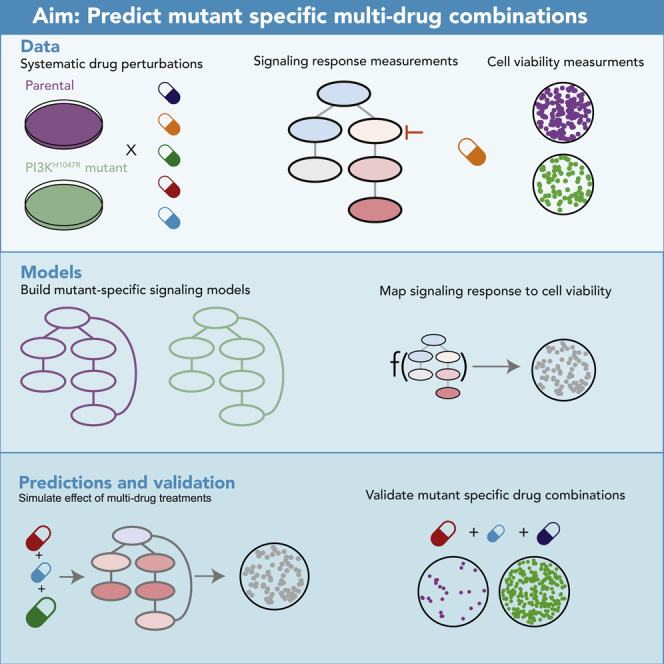

## Introduction

The dependency of tumors on activated signaling pathways results in therapeutic responses to inhibitors that block pathway activity (Weinstein, 2002). However, resistance to such targeted inhibitors inevitably develops (Holohan et al., 2013; Konieczkowski et al., 2018). Combinations of two targeted inhibitors can give more lasting clinical benefit, but resistance nonetheless emerges (Flaherty et al., 2012; Long et al., 2014). Combining more than two drugs might further extend the duration of the response (Boshuizen and Peeper, 2020), but toxicity becomes a major concern when multiple drugs are combined at their maximum tolerated dose. Recently, we found that partial inhibition of three or four kinases by combining multiple drugs at low dose (MLD) is surprisingly effective in receptor tyrosine kinase-driven tumors in multiple cancer types (Fernandes Neto et al., 2020). It prevents the development of resistance, and it is well tolerated by mice. Others have also shown the potential of multi-drug (low-dose) combinations in pre-clinical (Ryan et al., 2019; Ozkan-Dagliyan et al., 2020; Zoetemelk et al., 2020; Caumanns et al., 2019) and clinical (Van Cutsem et al., 2018, 2019) settings.

These findings warrant further exploration of multi-drug combination strategies. This will require a systematic way to explore promising drug combination treatments, including optimizing the dosing of the different drugs. The combinatorial explosion of the search space — there are more than two million possible 4-way combinations of the 89 (as of 2020 (Zhong et al., 2021)) FDA-approved targeted inhibitors, and 24 billion if each drug is to be tested at 10 different concentrations — means that *in vitro* testing of all combinations is infeasible. Computational approaches are required to prioritize promising combinations.

Recently, Nowak-Sliwinska and collaborators presented a “Feedback Systems Control” approach to explore the search space of possible multi-drug combinations (Nowak-sliwinska et al., 2016; Weiss et al., 2015; Zoetemelk et al., 2020). While this approach is promising, the method does not optimize for selectivity and the obtained results lack a mechanistic underpinning, making it hard to assess to what extent the results will generalize. Another promising approach is building mathematical models of cellular signaling, based on a limited set of perturbation experiments (Bosdriesz et al., 2018; Dorel et al., 2018; Klinger et al., 2013; Halasz et al., 2016; Saez-Rodriguez et al., 2009; Jastrzebski et al., 2018; Kirouac et al., 2013; Nyman et al., 2020). However, current approaches suffer from two major shortcomings. First, only a very limited number of such modeling approaches focus on the difference between cells with different mutation profiles (Bosdriesz et al., 2018; Saez-Rodriguez et al., 2011), which is critical for optimizing selectivity. Second, how inhibition of oncogenic signaling affects cell viability and specifically to what extent short-term signaling response is informative for longer term cell fate remain underexplored (Kirouac et al., 2013; Korkut et al., 2015; Nyman et al., 2020).

**Figure 1:**
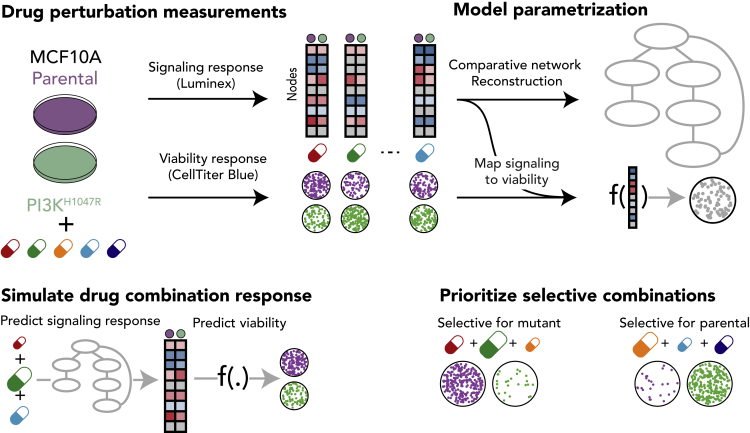
Overview of pipeline to prioritize promising selective low-dose multi-drug combinations **Top:** MCF10A parental and PI3K^H1047R^ cells are treated with inhibitors targeting the MAPK and AKT pathways. The signaling and cell viability responses are measured and used to build mutant-specific models of signal transduction networks and to parametrize the relationship between signaling response and cell viability. **Bottom:** These models are used to simulate the response to unobserved multi-drug combinations, at arbitrary concentrations, of the signaling networks and how this affects cell viability. In this way, low-dose multi-drug combinations that are likely selective for a particular cell line can be prioritized.

We therefore set out to establish and validate a combined experimental and computational pipeline to prioritize multi-drug combinations and their dosing based on mathematical models of drug response (Figure 1). Importantly, we aimed to find combinations that are selective for cells with a particular oncogenic driver mutation. To isolate the effect of the mutation, we used an isogenic cell line pair with and without a mutation. Specifically, we used MCF10A, a cell line derived from epithelial breast tissue (Soule et al., 1990), and an isogenic clone with the activating PI3K^H1047R^ mutation knocked in under its endogenous promoter (Di Nicolantonio et al., 2008). We measured the response of the MAPK and AKT pathway and cell viability after drug perturbations, and used the measurements to build mutant-specific signaling networks models using Comparative Network Reconstruction, a method we recently developed (Bosdriesz et al., 2018). In addition, we found that a non-linear model combining the response of phospho-ERK and phospho-AKT is highly predictive for cell viability, despite the fact that signaling response and cell viability are measured on completely different timescales of hours and days, respectively. Combining the signaling and viability models allowed us to simulate the effect of any multi-drug combination at any concentration and thus to prioritize promising combinations. Our models indicated that no combination of the drugs tested in this study would likely be selective for the PI3K mutant cells. To nonetheless validate our computational approach, we proceeded to predict which low-dose, multi-drug combinations were likely to be anti-selective, i.e. reduce the viability of the parental cells more strongly than that of the PI3K mutants. Experimental validation showed that 25 of the 30 combinations that we predicted to be anti-selective indeed had a significantly stronger effect in the parental cells than in the PI3K mutant cells.

## Results

### The signaling and viability response to drug perturbations in MCA10A parental and PI3K^H1047R^-mutated cells

**Figure 2:**
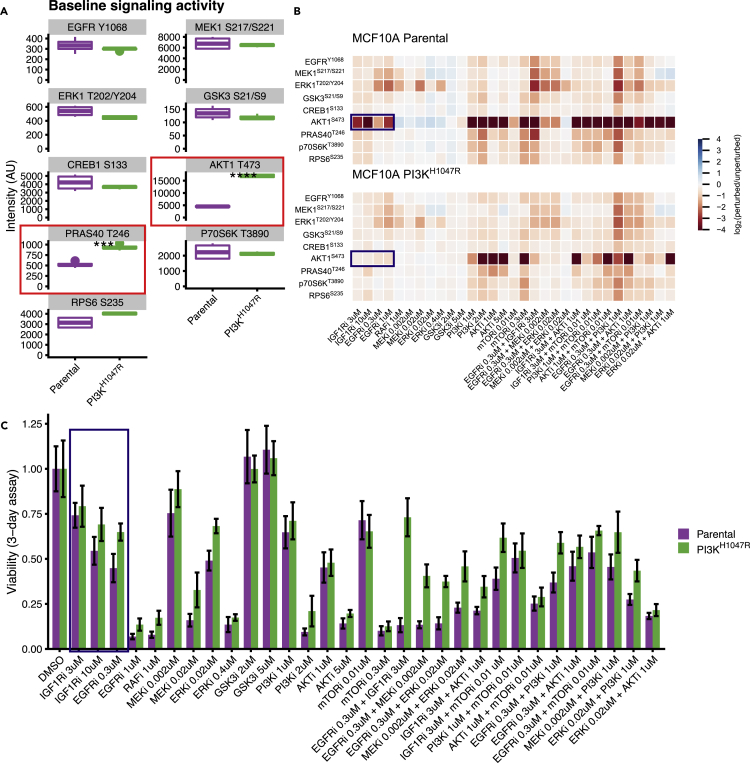
Profiling signaling and viability response of MCF10A parental en PI3K^H1047R^ cells to drug perturbations (A) Node activity in the unperturbed cells. Most nodes have similar activity in the parental and PI3K^H1047R^ cells, except AKT and PRAS40 (highlighted) which are downstream of PI3K. (B) Heatmap representing log_2_ fold changes of the signaling nodes upon drug perturbation compared to DMSO controls. The response of the parental and PI3K^H1047R^ cells is highly correlated, with some exceptions such that of AKT1 upon growth factor receptor inhibition (highlighted). Signaling response is measured after 2 h of drug treatment. The color scale is capped between −4 and 4 for visualization purposes. (C) Cell viability under the same drug treatments as reported in panel B. Both cell lines show a similar response profile. The strong differences in AKT response to growth factor receptor inhibition translate into mild differences in cell viability (highlighted). Cell viability is measured after 3 days of drug treatment. Error bars represent standard deviations.

To test how oncogenic mutations affect signal transduction networks and their downstream effects on cellular phenotypes such as cell viability, we used the MCF10A cell line (Soule et al., 1990) and an isogenic clone with the activating PI3K^H1047R^ mutation knocked in under its endogenous promoter (Di Nicolantonio et al., 2008). As expected, in the PI3K^H1047R^ cells, the baseline signaling activity of AKT and PRAS40, both downstream of PI3K, is elevated, but the other signaling nodes do not show significant differences in activity (Figure 2A). In the absence of drug perturbations, PI3K^H1047R^ mutant MCF10A cells have a comparable growth rate as their parental cells (Di Nicolantonio et al., 2008). Dose-response curves of selected PI3K and the MAPK pathway inhibitors showed subtle differences in sensitivities between the parental and the mutant cells (Figure S1A).

To study how the signaling networks of these cells respond to drug perturbations, and if the PI3K^H1047R^ mutation influences this, we perturbed both cell lines with inhibitors of the PI3K and MAPK pathways, and selected 2-drug combinations of these. Single drugs were tested at two different concentrations, corresponding roughly to their IC_50_ and IC_90_ values (except RAFi, which was only tested at IC_90_) and drug combinations were tested with both drugs at their IC_50_ values, to obtain a total of 34 different perturbations. We measured the response after 2 h of drug treatment (log_2_ fold change relative to DMSO control) of nine main nodes in the PI3K and MAPK signaling pathway using a multiplexed luminex assay to obtain more than 600 signaling drug-response measurements (Figure 2B, Tables S1, and S2). We selected the 2 h time point because this is the timescale for phospho-AKT (pAKT) to reach quasi steady state after PI3K pathway inhibition (Korkola et al., 2015) (Figure S1B). Luminex quantification showed excellent concordance with Western blots (Figure S1C). In addition, we measured the effect on cell viability using CellTiter Blue (Figure 2C, Tables S3, and S4). Generally, the differences in both signaling response and cell viability between the parental and PI3K mutant cells were subtle but consistent. For instance, while the responses of the signaling nodes of the parental and PI3K^H1047R^ cells are strongly correlated (Figures 2B and S1D), in the parental cells pAKT shows a strong negative response to growth factor receptor inhibition (EGFRi or IGF1Ri) that is nearly absent in the PI3K^H1047R^ mutant cells (Figure 2B, highlighted). However, this results in only mild differences in cell viability between the cell lines (Figure 2C, highlighted).

### Network reconstructions identify relevant differences between parental and PI3K mutant cells

To establish how the PI3K^H1047R^ mutation affects the signal transduction network, we used the drug-response measurements to perform Comparative Network Reconstruction (CNR) (Bosdriesz et al., 2018) of the MAPK and AKT pathways of both cell lines. CNR is a method that we have recently developed to reconstruct and quantify signaling networks and identify the most important quantitative differences between two or more cell lines. Prior knowledge about the network topology can be included, but the algorithm can also propose edges to be added to the network. The edge-weights are interpreted as the percent change in the downstream node activity in response to a 1% change in activity of the upstream node. Importantly, by penalizing differences between cell line models, CNR identifies which edges are quantitatively different between the two cell lines.

**Figure 3:**
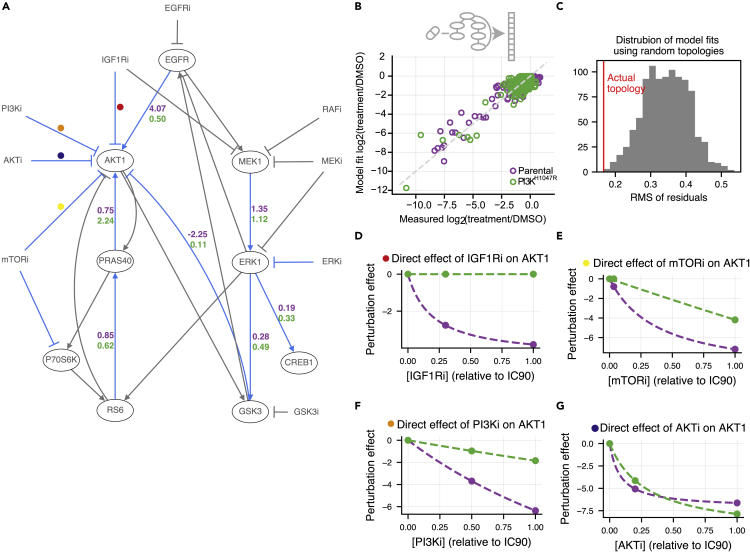
Mutant-specific network reconstructions show expected differences (A) Comparative Network Reconstruction (CNR) of MCF10A parental and PI3K^H1047R^ cells. Edges and direct perturbation effects that differ between the two cell lines are highlighted in blue. Gray edges do not differ between the cell lines. Edge strengths of the differing edges are represented by the purple (parental) and green (PI3K^H1047R^) numbers. Ovals indicate nodes. As expected, the most and the strongest differences between the cell lines are located close to AKT in the network (note that PI3K is not measured). (B) Comparison of network model fit with measured signaling response shows that the network model can explain the signaling response data well (Pearson correlation = 0.91). (C) Distribution of the root-mean-square (RMS) of residuals of models optimized using a random topology (gray), compared to that of the actual model used (red). All 1000 random topology models had the same number of edges as the actual model, and for all 1000, the fit was worse than for the actual model. (D–G) The estimated direct effect of IGF1R (C), mTOR (D), PI3K (E), and AKT (F) inhibition on AKT activity as a function of applied inhibitor concentration. Points indicate the estimated effects of the concentrations used in the CNR reconstruction, the dashed lines indicate the interpolated curves between these points (cf. STAR Methods, Equation 5). IGF1R, PI3K, and mTOR inhibition were modeled as directly affecting AKT because their actual targets were not measured.

We used the canonical MAPK and PI3K pathway interactions as prior information, and added four edges that were proposed by the CNR algorithm based on hyperparameters selected in a leave-one-out cross-validation loop (Figures 3A and S2A). The targets of some inhibitors were not measured in our panel. These were modeled as affecting the first downstream target that was measured. For instance, since both IGF1R and PI3K are not measured in our panel, IGF1R inhibition was modeled as targeting AKT1 directly. The model gave a good fit to the data (Pearson correlation = 0.91) (Figure 3B). To assess the significance of this fit, we compared the residuals of the model to 1000 models with the same number of randomly selected edges. Each of these 1000 random models had a worse fit than our model (p<0.001, Figure 3C). Using node-label permutation to the same overall distribution of ingoing and outgoing edges in the randomized networks gives similar results (Figure S2C).

CNR aims to identify the most relevant differences between cell lines by penalizing quantitative differences. These differences can be either the edges in the network, or the strength of inhibition of a drug to its direct target. This way, we identified 13 relevant differences between the parental and PI3K^H1047R^ cells. These differences are highlighted in blue in Figure 3A. The numbers next to the edges indicate the edge-strengths of the parental (purple) and PI3K^H1047R^ (green) cells. (For visualization purposes, only the strength of edges that differ between the cell lines is indicated. For full model visualization, see Figure S2B). The differences in target-inhibition strength between the cell lines are shown in Figures 3D–3G and S2E. We assessed the significance of the identified differences by comparing the residuals of our model to that of 1000 models with the same number of randomly selected differences. None of the random models had a better fit to the data (Figure S2D), indicating that the identified differences are, indeed, the most relevant differences.

As expected, most of the identified differences are located close to AKT in the network (Figure 3A, note that PI3K is not measured). Specifically, in the PI3K^H1047R^ cells, AKT is less sensitive to changes in EGFR and unresponsive to IGF1R inhibition (Figures 3A and 3D), which is consistent with PI3K being constitutively activated. Additionally, AKT is less responsive to PI3K and mTOR inhibition (Figures 3E and 3F). At the IC_50_, AKT is also less sensitive to AKT inhibition, but when AKTi is applied at its IC_90_, the PI3K^H1047R^ cells show a larger response (Figure 3G). This last observation might be explained by the higher baseline AKT activity of PI3K^H1047R^ cells, since if AKT activity is reduced to a similar absolute level, the log_2_ fold change of AKT in the mutant is higher.

In order to predict the signaling response to drugs combined at arbitrary concentrations, we parameterized the relation between target inhibition and drug concentration using the direct target-inhibition estimates for drug *k* on node *i* for the IC_50_ and IC_90_ that we obtained from the network reconstructions (cf. STAR Methods, Equation 5). The dashed lines in Figures 3D–3G and S2E indicate the curves that we parametrized in this way.

### Short-term signaling response is informative for long-term cell viability

**Figure 4:**
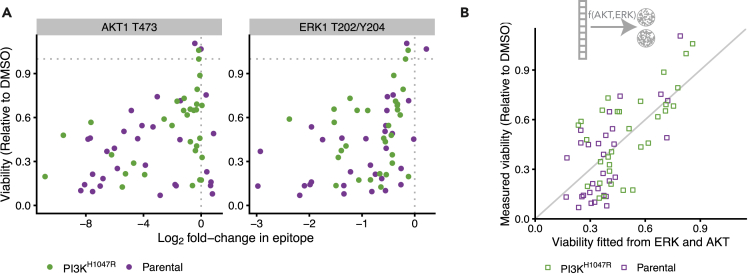
Short-term signaling response is predictive for longer term cell viability (A) Scatterplot of cell viability against log_2_ fold changes in AKT (left panel) and ERK (right panel) activity in response to drug treatments. The Pearson correlations are 0.36 and 0.42, respectively. (B) Scatterplot of model fit against measured cell viability based on a model where both ERK and AKT response are used to explain cell viability (cf. STAR Methods, Equation 1). The Pearson correlation between fit and measurement is 0.71.

To prioritize multi-drug combinations, the short-term response of the signaling network to a drug perturbation needs to be related to its longer term effect on cell viability. Important open questions here are: Is the short-term signaling response predictive to longer term cell viability? If so, which signaling outputs are most predictive, and what is their relation? The associations between the individual node-responses and cell viability were moderate even for the most strongly associated nodes, phospho-AKT (pAKT) and phospho-ERK (pERK), which had a Pearson correlation with cell viability of 0.36 and 0.42, respectively (Figure 4A). The responses of all other nodes also correlated somewhat with cell viability (Figure S3), but clearly no single node is a good predictor for cell viability.

We therefore investigated whether a model combining the response of multiple nodes described the cell viability data better. To this end, we first fitted a linear model using all nodes as predictors Equation 4a. This model gave a reasonable fit, but only the coefficients for pERK, pMEK, and pAKT were significant. The coefficient of pMEK had an unexpected positive sign, indicating that in this model a decrease in pMEK increases the predicted viability. In addition, the response of pMEK and pERK is highly correlated (Pearson correlation 0.81), making the contribution of pMEK to viability in this multivariate model hard to interpret. Next, we tried a linear model using only pERK and pAKT as predictors Equation 4b. However, this gave a worse fit in a leave-one-out cross-validation loop (Table 1).

**Table 1:** Comparison of the goodness of fit of functions relating signaling response to cell viability

Function	Type	σ (Model Fit)	L_2_-Norm (LOOCV)
$v_{k}∼1/\left(1-\frac{R_{AKT,k}}{K_{M,AKT}}-\frac{R_{ERK,k}}{K_{M,ERK}}\right)$	Non-linear	0.20	0.042
$v_{k}∼1/\left[\left(1-\frac{R_{AKT,k}}{K_{M,AKT}}\right)\cdot \left(1-\frac{R_{ERK,k}}{K_{M,ERK}}\right)\right]$	Non-linear	0.20	0.045
$v_{k}∼3/\left(1+2^{-\frac{R_{ERK,k}}{K_{M,ERK}}}+2^{-\frac{R_{AKT,k}}{K_{M,AKT}}}\right)$	Non-linear	0.21	0.048
$v_{k}∼4/\left[\left(1+2^{-\frac{R_{ERK,k}}{K_{M,ERK}}}\right)\cdot \left(1+2^{-\frac{R_{AKT,k}}{K_{M,AKT}}}\right)\right]$	Non-linear	0.21	0.048
$1-v_{k}∼\underset{i\in \text{nodes}}{\sum }{\text{β}}_{i}\cdot R_{ik}$	Linear	0.21	0.061
$1-v_{k}∼{\text{β}}_{AKT}\cdot R_{AKT,k}+{\text{β}}_{ERK}\cdot R_{ERK,k}$	Linear	0.28	0.093

σ is the mean residual standard error of the model fitted to the full data. The L_2_-norm is calculated over predictions made in a leave-one-out cross-validation loop. The non-linear models predict viability v from the log_2_-fold change of pERK and pAKT (R_ERK_ and R_AKT_) whereas the linear models fit the inhibition (1 - v). The table is ordered from best to worst fit.

We noticed that for both linear models there is a clear structure in the relation between the residuals and the fitted values (Figures S4A and S4B). This indicates that a non-linear model might be more suitable. To test this, we fitted a number of biologically motivated non-linear models relating the combined response of pAKT and pERK to cell viability. We selected pAKT and pERK based on their biological plausibility and because the analysis above indicated that of the nodes that we have measured, these are the ones that most strongly associated with cell viability. All models that we tested have the property that cell viability goes to zero if either pERK or pAKT is fully inhibited (cf. STAR Methods, (Equation 4c), (Equation 4d), (Equation 4e), (Equation 4f)), reflecting the biological assumption that both ERK and AKT activation are required for cell survival and growth.

To select the best model, we compared the standardized residuals of the model fits and the L2-norm of the residuals in a leave-one-out cross-validation loop of the different models (Table 1). All non-linear models had clearly better performance than the linear models, despite having equal or less free parameters. While overall the predictions of the different non-linear models were fairly similar (with Pearson correlations between their predictions between 0.92 and 0.99, Figure S4D), a Michaelis-Menten-like model of the form

(Equation 1) $$v_{k}=\frac{1}{1-\frac{R_{AKT,k}}{K_{M,AKT}}-\frac{R_{ERK,k}}{K_{M,ERK}}}$$

had the overall best performance on both metrics and shows no clear structure in the residuals (Figure S4C). Here, $v_{k}$ is the cell viability and $R_{ERK,k}$ and $R_{AKT,k}$ are the log_2_ fold changes of pERK and pAKT upon drug treatment *k*. The parameters $K_{M,ERK}$ and $K_{M,AKT}$ can be interpreted as the log_2_ fold changes of pERK and pAKT that cause 50% inhibition of cell viability. They differ slightly between the two cell lines, but the bootstrapped 95% confidence intervals strongly overlap (Figure S4E), so we do not want to overinterpret these differences. Importantly, this model gave a good fit to the data (Figure 4B), with a Pearson correlation between fitted and measured viability of 0.71.

Together, these results indicate that short-term signaling response is informative for longer term drug response, that pAKT and pERK are the most informative readouts of the nodes that we measured in our assay, and that the relation between signaling response and viability is non-linear.

### Prediction and validation of selective multi-drug combinations

**Figure 5:**
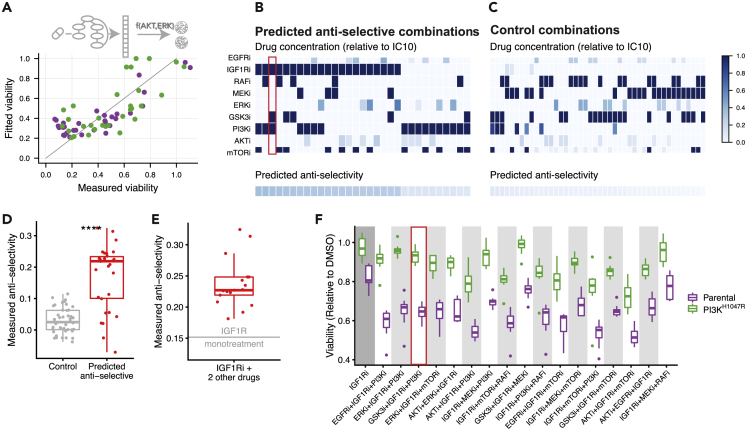
Experimental validation of anti-selective drug combination predictions (A) Scatterplot comparing full model fit (network model combined with signaling response-viability mapping) to the training data. The Pearson correlation between fit and measurement is 0.78. (B and C) Overview of drug combinations that we predicted to be anti-selective (B) and non-selective (C) based on this model. Drug concentrations are color-coded relative to their IC_10_. The bottom row indicates predicted anti-selectivity (defined as the difference in viability *v* between PI3K^H1047R^ and parental cells, i.e. $v^{\text{PI}3\text{K}-\text{H}1047\text{R}}-v^{\text{Parental}}$) of the combination. These combinations were subsequently tested in the validation experiments. (D) Boxplot comparing the measured anti-selectivity of the drug combinations predicted to anti-selective (panel B) or non-selective (panel C). Each point represents the mean anti-selectivity of one drug combination, which was each tested in eight replicates. The difference is highly significant (Wilcoxon signed-rank test $p<10^{-7}$). (E) Comparison of the measured anti-selectivity of IGFRi mono treatment, indicated by the horizontal gray line, with the selected IGF1Ri-containing 3-drug combinations (red boxplot). IGF1Ri-containing combinations are significantly more anti-selective than IGF1Ri mono treatment (one-sample t-test $p<10^{-7}$). (F) Boxplots comparing cell-viability of parental and PI3K^H1047R^ cells of the 11 (out of 17) IGF1Ri-containing drug combinations that are significantly more anti-selective than IGF1Ri mono treatment.

Next, we combined the network models (Figure 3A) with the parametrization of the signaling-viability model (Equation 1) to simulate the effect of unseen 3-drug combination at unseen drug concentrations on cell viability. When applying this model to the training data, the Pearson correlation between measured and fitted cell viability was 0.78 (Figure 5A). We used this model to prioritize multi-drug combinations and their dosing that maximize the selectivity, defined as the difference in viability *v* between parental and PI3K^H1047R^ cells: $v^{\text{Parental}}-v^{\text{PI}3\text{K}-\text{H}1047\text{R}}$.

To do this, for all possible 3-drug combinations, we optimized the concentrations such that the viability of the PI3K^H1047R^ mutants is minimized, under the constraint that the viability of the parental cells remains above 0.8 relative to DMSO control (cf. STAR Methods, Equation 7). To look for low-dose drug combinations, we added the constraint that each drug can be used maximally at its IC_10_. However, no drug combination was predicted to be selective for the PI3K^H1047R^ cells at any combination of concentrations. Since none of the single drugs shows selectivity toward the PI3K^H1047R^ cells (Figure 2C), this is not very surprising. Moreover, our network reconstructions indicated that the main effect of the PI3K^H1047R^ mutation is to render the MCF10A parental line independent of growth factor stimulation. Indeed, when we grew parental and PI3K^H1047R^ cells in the media without growth factor, this is what we observed (Figure S5).

To nonetheless validate our computational approach, we then looked for drug combinations that we predicted to be anti-selective, where anti-selectivity is defined as $v^{\text{PI}3\text{K}-\text{H}1047\text{R}}-v^{\text{Parental}}$. In our optimizations, we found 30 such combinations with an anti-selectivity > 0.1 (Figure 4B). Interestingly, IGF1R inhibition was part of all of the 17 combinations that we predict to be most anti-selective, while its anti-selectivity in the training data was only modest (Figure 2C). However, the difference in signaling response, and specifically pAKT, was much more pronounced (Figure 2B), and this latter aspect gets picked up in the network reconstructions (Figure 3A). A particularly interesting example is the combination IGF1Ri + PI3Ki + GSK3i. Here, both PI3Ki and GSK3i at their lower dose (IC_50_) show no anti-selectivity, yet this combination is predicted to be one of the most anti-selective ones. (Figure 5B, highlighted). As a control, we also selected 44 combinations that we predicted to be non-selective for either cell line (Figure 5C, cf. STAR Methods, Equation 8). A conservative power analysis, based on the accuracy of the viability predictions and the effect size of the anti-selectivity predictions, indicated a power of 90% to detect an overall difference in selectivity between the anti-selective and control combinations.

We then treated the parental and PI3K^H1047R^ cells with the 30 predicted to be anti-selective and 44 control combinations and measured their viability (Table S5). Combinations that we predicted to be anti-selective were indeed so, and this was highly significant when compared to the non-selective control combinations (Wilcoxon signed-rank test $p<10^{-7}$, Figure 5D). Individually, 25 of the 30 combinations predicted to be anti-selective were indeed significantly so (one-sided t-test p<0.05, Table S6).

As mentioned above, of the 30 predicted-to-be-*anti*-selective combinations we tested, 17 contain the IGF1R inhibitor, which is also mildly anti-selective as monotherapy. (None of the other inhibitors showed anti-selectivity as a monotherapy at their IC_10_, Figure S6). To rule out the possibility that our result is mainly driven by the anti-selectivity of IGF1Ri monotherapy, we compared the 17 IGF1Ri-containing drug combinations with IGF1Ri monotherapy. Figure 5E shows that each of the IGF1Ri-containing combinations we tested (red boxplot) is more anti-selective than IGF1Ri treatment alone (indicated by horizontal gray line). This effect is highly significant (one-sample t-test $p<10^{-7}$). When looking at the individual drug combinations, we found that 11 of the 17 IGF1Ri-containing combination treatments are significantly more anti-selective than IGF1Ri monotherapy (one-sided t-test p<0.05, Figure 5E and Table S7). This also includes the IGF1Ri + PI3Ki + GSK3i combination highlighted above, which is the second most anti-selective combination when ranked by effect size.

These results indicate that our pipeline is capable of making an accurate prioritization of mutation-specific low-dose multi-drug combinations. Importantly, these predictions are not always obvious, and would not have been possible without the help of mathematical models of the signal transduction networks and their relation to cell viability.

## Discussion

In this study, we have shown that it is possible to predict which multiple low-dose (MLD) three-drug combinations are likely to have a mutant-specific impact based on a combination of single and two-drug high-dose drug-response measurements and mathematical modeling. We have used drug perturbation experiments to reconstruct, quantify and compare signal transduction networks of an isogenic cell line pair, and linked the responses of these networks to cell viability. No single signaling readout is highly predictive, but a non-linear model combining the response of pERK and pAKT gave a good fit. Importantly, this showed that the short-term signaling response is predictive for cell viability, which is measured in longer term experiments. Based on the so-obtained models, we were able to predict combinations that are specifically effective in one cell line but not another. 25 of the 30 of the combinations that we predicted to be anti-selective indeed were so in validation experiments, despite the fact that the differences between the cell lines in the training data were very subtle. It is to be expected that it will be easier to find mutation-specific drug combinations when the effect of a mutation on the signaling networks and on cell viability is stronger.

All of the most strongly anti-selective drug combinations we identified contained the IGF1R inhibitor, but as monotherapy low-dose IGF1Ri is only mildly anti-selective. More generally, which multi-drug combinations are most selective or anti-selective is often far from obvious. For instance, while in the training data PI3Ki and GSK3i at their lower dose (IC_50_) individually show no selectivity toward the parental cells, the combination IGF1Ri + PI3Ki + GSK3i is one of the most anti-selective drug combinations, both as predicted by our model and as measured by validation experiments. This underscores the need for mathematical modeling in prioritizing promising combinations.

We focused on a relatively small network of nine nodes centered around the MAPK and AKT signaling axis. However, technologies are emerging that will make it feasible to perform many perturbations and measure the signaling network responses in a highly multiplex manner. For instance, reverse-phase protein arrays have been used to quantify the response of hundreds of (phospho)-proteins to hundreds of drug perturbations (Korkut et al., 2015) in large cell line panels (Zhao et al., 2020), and van Buggenum et al. used DNA-barcoded antibodies to quantify the response of 70 (phospho)-proteins to ∼300 different kinase inhibitors (van Buggenum et al., 2018). By leveraging such technologies, the pipeline developed here could be used to prioritize drug combinations targeting the whole spectrum of signaling pathways.

In conclusion, here we have shown that it is feasible to make accurate, non-trivial predictions about (anti-)selectivity of multi-drug combinations based on mathematical models of signaling transduction networks. In combination with suitable model systems, this framework makes it possible to rationally design biomarker-selective low-dose multi-drug combinations.

### Limitations of the study

The main limitation of this study is that, according to our model, no combination of the drugs that we tested is likely to be selective, i.e. inhibit cells with an oncogenic PI3K^H1047R^ mutation more strongly than their parental counterparts, at least in this particular model system The absence of oncogene-specific sensitivities is presumably due to an absence of “oncogene addiction” (Weinstein, 2002) to the PI3K mutation in the PI3K^H1047R^ MCF10A cells. In the absence of drug treatment, the mutation has no effect on proliferation under the growth conditions we used, and this mutation therefore presumably does not induce any vulnerabilities in this cell line. Our network reconstruction suggests that the main effect of the PI3K^H1047R^ mutation on MCF10A cells is to make them growth factor-independent, consistent with previous observations (Gustin et al., 2009). We focused on targeting and modeling the AKT and MAPK pathways as these are most likely to show pronounced differences between the cell lines (Wu et al., 2014). While some proteins in alternative pathways are also impacted by the the PI3K^H1047R^ mutation (Wu et al., 2014), we believe that it is unlikely that alternative targets would show selectivity toward PI3K mutations, but we cannot fully exclude this possibility.

The inability to identify selective drug combinations is due to the particularities of the MCF10A isogenic cell line pair model, and not due to the computational model. While isogenic cell line pairs with a mutation knocked in are attractive models because they isolate the effect of the mutation, they may thus not always be the best model system to study oncogene-specific sensitivities. An alternative approach might be to use cancer cell lines of which one of the driver mutations is removed (Haagensen et al., 2016; Torrance et al., 2001; Martin et al., 2017). Alternatively, a larger, more heterogeneous panel of cell lines with and without a particular biomarker could be used (Jastrzebski et al., 2018; Klinger et al., 2013; de Lint et al., 2016; Iorio et al., 2016). In this scenario, one would look at commonalities in the signaling network response of the cell lines with the biomarker compared to the lines without it, and use this to propose combinations that are selective of the biomarker carrying cell lines. Finally, matched tumor and normal organoids from the same patient could be used for truly personalized models (Veninga and Voest, 2021).

To parametrize the relation between drug concentration and target inhibition (Equation 5), we interpolated the effects estimated at two different drug concentrations, using a simple model with just two free parameters. While this gives a reasonable indication about the relation between drug concentration and target inhibition, two data points are not enough to make strong inferences about the precise form of the function relation drug concentration to target inhibition, and whether this differs for different drugs. Similarly, we used a very simple model with just two free parameters to relate signaling response to cell viability. While this model gave a good fit to the data, more data are needed to precisely determine the form of this relationship. For instance, Figure 4B suggests that the model rarely predicts viabilities below ∼0.3. This might be due to the form of Equation 1 which approaches zero asymptotically slow with increasing inhibition of ERK or AKT activity. Since we were aiming to predict low-dose combinations that are (anti)selective, the range of viabilities of our model predictions was roughly between 0.4 and 1, so this does not affect our predictions strongly. Nonetheless, the fit might be improved with more complex models, for instance by including something akin to a Hill-coefficient in Equation 1. In future studies, performing drug perturbation experiments at more concentrations could help to better determine the precise relations between drug concentration and target inhibition and between signaling response and cell viability.

Targeted inhibitors always have off-target effects, but there are multiple reasons why these are unlikely to affect our main results. First, we focus on predicting differential drug response, and only *mutant-specific* off-target effects, which are unlikely to be dominant, will influence this. Furthermore, our predictions are based on modeling the on-target effects. If off-target effects would be dominant, these would likely not have validated. Finally, we combine the drugs at low concentrations, at which the selectivity of targeted drugs is typically high (Klaeger et al., 2017). Nonetheless, when in future studies selective combinations are found, it could be advisable to exclude off-target effects before proceeding to *in vivo* validation by testing alternative drugs for the same target.

## STAR★Methods

### Key resources table

**Table:** 

REAGENT or RESOURCE	SOURCE	IDENTIFIER
**Antibodies**		

CREB1^S133^ (Detection Mix)	ProtAtOnce Ltd.	Cat#PR1707253
EGFR^Y1068^ (Detection Mix)	ProtAtOnce Ltd.	Cat#PR1707253
ERK1^T202/Y204^ (Detection Mix)	ProtAtOnce Ltd.	Cat#PR1707253
GSK3^S21/S9^ (Detection Mix)	ProtAtOnce Ltd.	Cat#PR1707253
MEK1^S217/S221^ (Detection Mix)	ProtAtOnce Ltd.	Cat#PR1707253
p70RSK^T389^ (Detection Mix)	ProtAtOnce Ltd.	Cat#PR1707253
PRAS40^T246^ (Detection Mix)	ProtAtOnce Ltd.	Cat#PR1707253
RPS6^S235^ (Detection Mix)	ProtAtOnce Ltd.	Cat#PR1707253
AKT1^T473^	Bio-Rad	Cat#171V50001M

**Chemicals, peptides, and recombinant proteins**		

EGFRi (Gefitinib)	MedKoo Biosciences	Cat#100140
IGF1Ri (OSI-906)	MedKoo Biosciences	Cat#202096
RAFi (LY3009120)	MedKoo Biosciences	Cat#206161
MEKi (Trametinib)	MedKoo Biosciences	Cat#201458
ERKi (SCH772984)	MedKoo Biosciences	Cat#406578
GSK3i (3F8)	MedKoo Biosciences	Cat#564605
PI3Ki (BKM120)	MedKoo Biosciences	Cat#204690
AKTi (MK-2206)	MedKoo Biosciences	Cat#577522
mTORi (AZD8055).	MedKoo Biosciences	Cat#200312

**Critical commercial assays**		

CellTiter-Blue	Promega Corporation	Cat#G8081
Cell Signaling Reagent Kit	BioRad	Cat#171304006M
9 plex BEAD MIX	ProtAtOnce	Cat#PR1707252

**Deposited data**		

Luminex data	This paper	Zenodo: https://doi.org/10.5281/zenodo.6594812 and https://github.com/evertbosdriesz/cnr-selective-combos
Envision quantification	This paper	Zenodo: https://doi.org/10.5281/zenodo.6594812 and https://github.com/evertbosdriesz/cnr-selective-combos

**Experimental models: Cell lines**		

MCF10A Parental	Horizon Discovery	Cat#HD PAR-003
MCF10A PI3K^H1047R^	Horizon Discovery	Cat#HD 101-011

**Software and algorithms**		

Original code and notebooks	This paper	Zenodo: https://doi.org/10.5281/zenodo.6594812 and https://github.com/evertbosdriesz/cnr-selective-combos
Comparative Network Reconstruction	Bosdriesz et al., (2018)	https://github.com/NKI-CCB/cnr
MixedIC50	Vis et al., (2016)	https://github.com/NKI-CCB/MixedIC50
ILOG CPLEX Optimization Studio (version 12.8)	IBM	N/A
Mathematica (version 12.0)	Wolfram Research	N/A

**Other**		

EnVision	PerkinElmer	N/A
Bio-Plex Protein Array system	Bio-Rad	N/A

### Resource availability

#### Lead contact

Further information and requests for resources and reagents should be directed to and will be fulfilled by the lead contact, Evert Bosdriesz (e.bosdriesz@vu.nl).

#### Materials availability

This study did not generate new unique reagents.

### Experimental model and subject details

#### Cells and cell culture

Human parental and PI3K^H1047R^^/+^ MCF10A cell lines were obtained from Horizon discovery (HD PAR-003 and HD 101-011). Cells were cultured in DMEM/F-12 including 2.5 mM L-glutamine and 15 mM HEPES, supplemented with 5% horse serum, 10 μg/mL insulin, 0.5 μg/mL hydrocortisone and 0.1 μg/mL cholera toxin. Mycoplasma tests were performed every 2 months.

#### Reagents and compounds

The following inhibitors were used in this study: EGFRi (Gefitinib), IGF1Ri (OSI-906), RAFi (LY3009120), MEKi (Trametinib), ERKi (SCH772984), GSK3i (3F8), PI3Ki (BKM120), AKTi (MK-2206), mTORi (AZD8055). All inhibitors were purchased from MedKoo Biosciences. The luminex antibodies against CREB1^S133^, EGFR^Y1068^, ERK1^T202/Y204^, GSK3^S21/S9^, MEK1^S217/S221^, p70RSK^T389^, PRAS40^T246^ and RPS6^S235^ were purchased from ProtATonce Ltd. The luminex antibody against AKT1^T473^ was purchased from BioRad.

### Method details

#### Drug perturbation and validation experiments

All the cell-viability measurements were performed in biological triplicates, each with 2 technical replicates, using black-walled 384-well plates (Greiner 781091). Cells were plated at the optimal seeding density (200 cells per well) and incubated for approximately 24 hours to allow attachment to the plate. Drugs were then added to the plates using the Tecan D300e digital dispenser. 10 μM phenylarsine oxide was used as positive control (0% cell viability) and DMSO was used as negative control (100% cell viability). Three days later, culture medium was removed and CellTiter-Blue (Promega G8081) was added to the plates. After 2 hours incubation, measurements were performed according to manufacturer’s instructions using the EnVision (PerkinElmer). Viabilities were normalized per cell line according to $\left(\text{treatment}-{\text{PAO}}_{mean}\right)/\left({\text{DMSO}}_{mean}-{\text{PAO}}_{mean}\right)$. IC_50_ and IC_90_ values were fitted using the R-package MixedIC50 (Vis et al., 2016) (code available at https://github.com/NKI-CCB/MixedIC50).

The signaling response measurements were performed using 6-well plates (Greiner 657165). 300K cells per well were plated and incubated for approximately 24 hours to allow attachment to the plate. Drugs were then added to the plates and protein was harvested after 2 hours using the Bio-Plex Pro Cell Signaling Reagent Kit (BioRad 171304006M) according to the manufacturer’s instructions. Protein concentration of the samples was normalized after performing a Bicinchoninic Acid (BCA) assay (Pierce BCA, Thermo Scientific), according to the manufacturer’s instructions. Cell lysates were analyzed using the Bio-Plex Protein Array system (Bio-Rad, Hercules, CA) according to the suppliers protocol as described previously (Klinger et al., 2013). Intensities were normalized by subtracting blanks for each epitope and correcting for protein concentration.

### Quantification and statistical analysis

#### Comparative network reconstruction

MAPK and AKT signaling networks of the parental and PI3K^H1047R^ mutant cell lines were reconstructed based on the Luminex drug-response data using Comparative Network Reconstruction (CNR)(Bosdriesz et al., 2018). Briefly, CNR is a network reconstruction method based on Modular Response Analysis (Kholodenko et al., 2002). It links the matrix of measured node responses to a set of perturbations, $R^{x}$ (where $R_{ik}^{x}$ is defined as log_2_ fold change of node *i* in response to perturbation *k* in cell line *x*) to the matrix of unobserved interaction strengths $r^{x}$ (where $r_{ij}^{x}$ is the logarithmic partial derivative of node *i* with respect to node *j* in cell line *x*) and the direct perturbation effects $s^{x}$ (with $s_{ik}^{x}$ the scaled direct effect of perturbation *k* on node *i* in cell line *x*). These matrices are related through

(Equation 2) $$r^{x}\cdot R^{x}+-s^{x}=0,\;\forall x.$$

In principle, $r^{x}$ and the values of the elements in $s^{x}$ (the targets of the inhibitors are assumed to be known) can be obtained by solving this set of equations, but in practice the problem is often under-determined. CNR solves this problem by reformulating it as optimization procedure to find a model that balances data-fit with a model complexity by penalizing the number of edges (non-zero entries in r) and differences between cell lines (entries in r that are quantitatively different between the cell lines). The optimization problem reads:

(Equation 3) $$\begin{array}{ccc} \text{Minimize}: & \sum_{n}\sum_{i,j}\sum_{x}\varepsilon_{in}^{x2}+\eta \cdot I_{ij}^{\text{edge}}+\theta \cdot \left(I_{ij}^{\text{diff}}+I_{in}^{\text{sdiff}}\right) & \\ \text{Subject}\;\text{to}: & \sum_{k}r_{ik}^{x}\cdot R_{kn}^{x}+s_{in}^{x}=\varepsilon_{in}^{x} & \forall i,j,n,x\\ & I_{ij}^{\text{edge}}=0\Rightarrow r_{ij}^{x}=0 & \forall i,j,x\\ & I_{ij}^{\text{diff}}=0\Rightarrow r_{ij}^{x}-\;r_{ij}^{\text{mean}}=0 & \forall i,j,x\\ & I_{in}^{\text{sdiff}}=0\Rightarrow s_{in}^{x}-s_{in}^{\text{mean}}=0 & \forall i,n,x\\ & r_{ij}^{\text{mean}}=1/N_{\text{cell lines}}\underset{x}{\sum }r_{ij}^{x} & \forall i,j\\ & s_{in}^{\text{mean}}=1/N_{\text{cell lines}}\underset{x}{\sum }s_{in}^{x} & \forall i,n\\ & I^{\text{edge}},I^{\text{dif}},I^{\text{sdiff}}\in \left\{0,1\right\} & \\ & n\in \left\{\text{perturbations}\right\};i,j,k\in \left\{\text{nodes}\right\};x\in \left\{\text{parental},\text{PI}3{\text{K}}^{\text{H}1047\text{R}}\right\} & \end{array}$$

where the εs are the model residuals. Solving this optimization problem gives the matrices r and s for a given R.

Additional constraints reflecting the experimental design were added to the CNR problem.

• $s_{ik}$ is negative and stronger for higher drug concentrations, i.e. $0>s_{ik}\left(\left[IC_{50}\right]\right)>s_{ik}\left(\left[IC_{90}\right]\right)$.
• Each inhibitor-target pair has a single indicator for the difference in perturbations strengths for both inhibitor concentrations, i.e. if $I_{ik}^{\text{sdiff}}=0$, both constraints $s_{ik}^{\text{parental}}\left(\left[IC_{50}\right]\right)=s_{ik}^{\text{PI}3\text{K}}\left(\left[IC_{50}\right]\right)$ and $s_{ik}^{\text{parental}}\left(\left[IC_{90}\right]\right)=s_{ik}^{\text{PI}3\text{K}}\left(\left[IC_{90}\right]\right)$ are active.
• Most inhibitors are modelled as a perturbation to their direct target, i.e. EGFRi, MEKi, ERKi, GSK3i and AKTi are modelled as perturbations to EGFR, MEK1, ERK1, GSK3 and AKT1 respectively.
• The MEK inhibitor interferes not only with MEK phosphorylation, but also with its catalytic efficiency. Hence, MEK inhibition was additionally modelled as a perturbation to its downstream proteins (c.f. Bosdriesz et al. (2018)).
• Some inhibitors target kinases that were not measured in our assay. The effect of these inhibitors was modelled as a perturbation to the (canonical) downstream nodes of the kinases being inhibited. Specifically, IGF1R inhibition was modelled as a perturbation to MEK1 and AKT1, PI3K inhibition as a perturbation to AKT1, RAF inhibition as a perturbation to MEK1, and mTOR inhibition as a perturbation to AKT1 and p70S6K.

Prior information about network topology was included by setting the indicators of a set of canonical MAPK and PI3K pathway interactions $I_{ij}^{\text{edge}}=1$. These indicator constraints are added to the optimization problem described in Equation 3. The corresponding edge-strengths, together with those of edges that might be added to the network, are found by solving the optimization problem. Hyperparameter were set to η=0.1 and θ=2.0 based on a leave one out cross validation loop. Single drug treatments were not included in the leave one out cross validation because each drug concentration needs to be present in at least one perturbation to estimate the corresponding parameter. The final model was obtained by restricting the topology to the prior network information with addition of the 4 edges that were identified in the leave one out cross-validation loop, and then performing the optimization with θ=2.0.

The full Comparative Network Analysis can be found in the Jupyter notebook under the following link: https://github.com/evertbosdriesz/cnr-selective-combos/blob/master/python/cnr-mcf10a-pi3k.ipynb.

#### Randomized models

To obtain the distribution of residuals shown in Figure 3C, 1000 models with a random topology were generated by randomly selecting 16 (out of all possible 72) edges, setting the corresponding indicator to $I_{ij}^{\text{edge}}=1$, and setting the indicators of all other edges to zero. To focus on the effect of model topology only, we did not allow for any difference between the cell lines by setting θ to infinity using cplex.infinity. Subsequently, edge weights were obtained by solving the CNR optimization problem described in Equation 3, and the corresponding Root Mean Square (RMS) of residuals, defined as $\sqrt{\sum_{i,n,x}\varepsilon_{in}^{x\;2}}/N$, was calculated. To make a fair comparison, we also calculated the residual of our actual model without any differences between the cell lines. To this end, we set the indicators of the edges of the actual model $I_{ij}^{\text{edge}}=1$ and all others to 0, set θ=cplex.infinity, and re-optimized Equation 3.

Similarly, the distribution of residuals shown in Figure S2C was obtained by randomly permuting node-labels of the input data and optimizing the model with the same network topology as the actual model.

Finally, to obtain the distribution of residuals for random differences between the cell lines shown in Figure S2D, 1000 models with randomly selected $I^{\text{diff}}$ and $I^{\text{sdiff}}$ were generated. For all these models, the topology was first fixed to the topology of the actual model by setting the indicators $I_{ij}^{\text{edge}}=1$ for the edges that are present in the actual model, and all others to 0. Subsequently, 13 randomly selected indicator for differences between the cell lines $I^{\text{diff}}$ and $I^{\text{sdiff}}$ were set to 1, the others to 0, and the optimization in Equation 3 was solved using these constraints.

The relation between signaling output and cell viability

The viability (relative to DMSO control) upon perturbation *k*, $v_{k}$, were fitted to the following functions:

(Equation 4a) $$1-v_{k}=\sum_{i\;\in \;\text{nodes}}\beta_{i}R_{ik}$$

(Equation 4b) $$1-v_{k}=\beta_{AKT}\cdot R_{AKT,k}+\beta_{ERK}R_{ERK,k}$$

(Equation 4c) $$v_{k}=\frac{1}{1-\frac{R_{AKT,k}}{K_{M,AKT}}-\frac{R_{ERK,k}}{K_{M,ERK}}}$$

(Equation 4d) $$v_{k}=\frac{1}{1-\frac{R_{AKT,k}}{K_{M,AKT}}}\times \frac{1}{1-\frac{R_{ERK,k}}{K_{M,ERK}}}$$

(Equation 4e) $$v_{k}=\frac{3}{1+2^{-\frac{R_{ERK,k}}{K_{M,ERK}}}+2^{-\frac{R_{AKT,k}}{K_{M,AKT}}}}$$

(Equation 4f) $$v_{k}=\frac{2}{1+2^{-\frac{R_{ERK,k}}{K_{M,ERK}}}}\times \frac{2}{1+2^{-\frac{R_{AKT,k}}{K_{M,AKT}}}}$$

where $R_{AKT,k}$ and $R_{ERK,K}$ are the log_2_-fold changes of pAKT and pERK relative to DMSO control upon perturbation *k*, respectively. $K_{M,AKT}$ and $K_{M,ERK}$ are the parameters to be fitted for the nonlinear equations and can be interpreted as the $R_{AKT,k}$ and $R_{ERK,k}$ values for which the viability is reduced by 50% (or 25% and 33% for Equations 4e and 4f, respectively). Fitting was performed using the lm and nls functions of R (Core Team, 2021) for the linear and non-linear models, respectively. Mean residual standard errors (σ) were obtained using the sigma function. Leave one out cross-validation was performed on a per cell-line basis. Bootstraps were performed using the function bootstrap from the ‘rsample’ package (Kuhn and Wickham, 2020).

All code and details for this analysis can be found in the RMarkdown-file under the following link: https://github.com/evertbosdriesz/cnr-selective-combos/blob/master/R/02-perturbations/mapping-signaling-drugresponse.Rmd.

#### Multi-drug response simulations and prediction of selective 3-drug combinations

CNR gives an estimate of the direct target inhibition of each drug only for the concentrations at which the drug was applied. To be able to simulate the effect of unseen drug concentrations, the relations between the applied concentration of drug *k*, $\left[I_{k}\right]$, and target inhibition of node *i* in response to this, $s_{ik}$ were fitted to the following function for each inhibitor-target pair,

(Equation 5) $$s_{ik}\left(\left[I_{k}\right]\right)=\frac{I_{max,ik}\text{∗}\left[I_{k}\right]}{K_{I,ik}+\left[I_{k}\right]}.$$

The parameters $I_{max,ik}$ and $K_{I,ik}$ were fitted using the $s_{ik}$-values for $\left[I_{k}\right]=IC_{50}$ and $IC_{90}$ obtained from the CNR optimizations with the curve_fit function from the python ‘scipy.optimize’ package (Virtanen et al., 2020). For convenience all drug concentrations were normalized to the highest concentration applied (the $IC_{90}$). In all analyses only interpolations and not extrapolations are used (0≤[I]≤1).

$R_{A+B+C}$, the vector of simulated log_2_ fold changes in response to a perturbation with drugs *A*, *B* and *C*, at concentration $\left[I_{A}\right]$, $\left[I_{B}\right]$ and $\left[I_{C}\right]$ was calculated as

(Equation 6) $$R_{A+B+C}=r^{-1}\left(s_{A}\left(\left[I_{A}\right]\right)+s_{B}\left(\left[I_{B}\right]\right)+s_{C}\left(\left[I_{C}\right]\right)\right),$$

to obtain $R_{AKT,A+B+C}$ and $R_{ERK,A+B+C}$. These were then used to calculate viability according to Equation 4c. Together, this allows for simulating the effect on cell viability of drug combinations and concentrations that were not seen in the training data.

For each possible 3-drug combination, the (anti)-selectivity for cell line *x* relative to *y* was optimized by solving the following optimization problem:

(Equation 7) $$\begin{array}{cc} \text{Minimize}: & v_{A+B+C}^{x}\\ \text{Subject}\;\text{to}: & v_{A+B+C}^{y}\geq v^{y,min}\\ & 0<\left[I_{k}\right]<IC_{10}\;k\in \left\{A,B,C\right\} \end{array}$$

where $v^{y,min}$ is the cutoff used for the minimal viability that cell line *y* should have under the 3-drug treatment, and that we (somewhat arbitrarily) set to 0.8. Specifically, to optimize selectivity we use $x=PI3K^{\text{H}1047\text{R}},y=Parental$ and to optimize anti-selectivity we use $x=Parental,y=PI3K^{\text{H}1047\text{R}}$.

Similarly, unselective control combinations were obtained by solving the optimization problem:

(Equation 8) $$\begin{array}{ll} \text{Minimize}: & {\left(v_{A+B+C}^{x}-0.8\right)}^{2}+{\left(v_{A+B+C}^{y}-0.8\right)}^{2}\\ \text{Subject}\;\text{to}: & 0<\left[I_{k}\right]<IC_{10}\;k\;\in \;\left\{A,B,C\right\} \end{array}$$

for all possible 3-drug combinations.

A power analysis of the predictions was done by performing 1000 simulations with addition gaussian noise with a mean 0 and a standard deviation 0.25 (based on the residuals of our viability predictions) to the results, and counting in what fraction there was an observable difference between the two groups.

The optimizations were performed in Wolfram Mathematica (Wolfram Research, Inc., 2019) (version 12.0) using the NMinimize function. The full optimization and power analysis can be found in the Mathematica notebook under the following link: https://github.com/evertbosdriesz/cnr-selective-combos/blob/master/mathematica/optimize-combinations.nb.

## Data Availability

All data and code required to reproduce the results and figures in this paper are available at Zenodo. DOIs are listed in the key resources table. Alternatively, they can be directly accessed on GitHub at https://github.com/evertbosdriesz/cnr-selective-combos. All data produced in this study are included in the published article and its supplemental information, or are available from the lead contact upon request.

## Supplemental information

Document S1. Figures S1–S6

Table S1. Log_2_ fold changes of signaling nodes in response to drug treatment of MCF10A parental cells, related to Figure 2B

Table S2. Log_2_ fold changes of signaling nodes in response to drug treatment of MCF10A PI3KH1047R cells, related to Figure 2C

Table S3. Cell viability of MCF10A parental cells after drug treatment, related to Figure 2C

Table S4. Cell viability of MCF10A PI3KH1047R cells after drug treatment, related to Figure 2C

Table S5. Cell viability measurements of the anti-selective and control multi-drug combinations, related to Figure 5

Table S6. Statistical analysis of anti-selective multi-drug combinations, related to Figures 5D and 5F

Table S7. Comparison of the anti-selectivity of IGF1Ri mono-treatment to IGF1Ri containing multi-drug combinations, related to Figures 5E and 5F

## References

[bib1] Bosdriesz E., Prahallad A., Klinger B., Sieber A., Bosma A., Bernards R., Blüthgen N., Wessels L.F.A. (2018). Comparative Network Reconstruction using mixed integer programming. Bioinformatics.

[bib2] Boshuizen J., Peeper D.S. (2020). Rational cancer treatment combinations: an urgent clinical need. Mol. Cell.

[bib3] Caumanns J.J., van Wijngaarden A., Kol A., Meersma G.J., Jalving M., Bernards R., van der Zee A.G.J., Wisman G.B.A., de Jong S. (2019). Low-dose triple drug combination targeting the PI3K/AKT/mTOR pathway and the MAPK pathway is an effective approach in ovarian clear cell carcinoma. Cancer Lett..

[bib4] de Lint K., Poell J.B., Soueidan H., Jastrzebski K., Vidal Rodriguez J., Lieftink C., Wessels L.F.A., Beijersbergen R.L. (2016). Sensitizing triple-negative breast cancer to PI3K inhibition by cotargeting IGF1R. Mol. Cancer Ther..

[bib5] Di Nicolantonio F., Arena S., Gallicchio M., Zecchin D., Martini M., Flonta S.E., Stella G.M., Lamba S., Cancelliere C., Russo M. (2008). Replacement of normal with mutant alleles in the genome of normal human cells unveils mutation-specific drug responses. Proc. Natl. Acad. Sci. USA.

[bib6] Dorel M., Klinger B., Gross T., Sieber A., Prahallad A., Bosdriesz E., Wessels L.F.A., Blüthgen N. (2018). Modelling signalling networks from perturbation data. Bioinformatics.

[bib7] Fernandes Neto J.M., Nadal E., Bosdriesz E., Ooft S.N., Farre L., McLean C., Klarenbeek S., Jurgens A., Hagen H., Wang L. (2020). Multiple low dose therapy as an effective strategy to treat EGFR inhibitor-resistant NSCLC tumours. Nat. Commun..

[bib8] Flaherty K.T., Robert C., Hersey P., Nathan P., Garbe C., Milhem M., Demidov L.V., Hassel J.C., Rutkowski P., Mohr P., METRIC Study Group (2012). Improved survival with MEK inhibition in BRAF-mutated melanoma. N. Engl. J. Med..

[bib9] Gustin J.P., Karakas B., Weiss M.B., Abukhdeir A.M., Lauring J., Garay J.P., Cosgrove D., Tamaki A., Konishi H., Konishi Y. (2009). Knockin of mutant PIK3CA activates multiple oncogenic pathways. Proc. Natl. Acad. Sci. USA.

[bib10] Haagensen E.J., Thomas H.D., Mudd C., Tsonou E., Wiggins C.M., Maxwell R.J., Moore J.D., Newell D.R. (2016). Pre-clinical use of isogenic cell lines and tumours in vitro and in vivo for predictive biomarker discovery; impact of KRAS and PI3KCA mutation status on MEK inhibitor activity is model dependent. Eur. J. Cancer..

[bib11] Halasz M., Kholodenko B.N., Kolch W., Santra T. (2016). Integrating network reconstruction with mechanistic modelling to predict cancer therapy. Sci. Signal..

[bib12] Holohan C., Van Schaeybroeck S., Longley D.B., Johnston P.G. (2013). Cancer drug resistance: an evolving paradigm. Nat. Rev. Cancer.

[bib13] Iorio F., Knijnenburg T.A., Vis D.J., Bignell G.R., Menden M.P., Schubert M., Aben N., Gonçalves E., Barthorpe S., Lightfoot H. (2016). A landscape of pharmacogenomic interactions in cancer. Cell.

[bib14] Jastrzebski K., Thijssen B., Kluin R.J.C., de Lint K., Majewski I.J., Beijersbergen R.L., Wessels L.F.A. (2018). Integrative modeling identifies key determinants of inhibitor sensitivity in breast cancer cell lines. Cancer Res..

[bib15] Kholodenko B.N., Kiyatkin A., Bruggeman F.J., Sontag E., Westerhoff H.V., Hoek J.B. (2002). Untangling the wires: a strategy to trace functional interactions in signaling and gene networks. Proc. Natl. Acad. Sci. USA.

[bib16] Kirouac D.C., Du J.Y., Lahdenranta J., Overland R., Yarar D., Paragas V., Pace E., McDonagh C.F., Nielsen U.B., Onsum M.D. (2013). Computational modeling of ERBB2-amplified breast cancer identifies combined ErbB2/3 blockade as superior to the combination of MEK and AKT inhibitors. Sci. Signal..

[bib17] Klaeger S., Heinzlmeir S., Wilhelm M., Polzer H., Vick B., Koenig P.A., Reinecke M., Ruprecht B., Petzoldt S., Meng C. (2017). The target landscape of clinical kinase drugs. Science (New York, N.Y.).

[bib18] Klinger B., Sieber A., Fritsche-Guenther R., Witzel F., Berry L., Schumacher D., Yan Y., Durek P., Merchant M., Schäfer R. (2013). Network quantification of EGFR signaling unveils potential for targeted combination therapy. Mol. Syst. Biol..

[bib19] Konieczkowski D.J., Johannessen C.M., Garraway L.A. (2018). A convergence-based framework for cancer drug resistance. Cancer Cell.

[bib20] Korkola J.E., Collisson E.A., Heiser L., Oates C., Bayani N., Itani S., Esch A., Thompson W., Griffith O.L., Wang N.J. (2015). Decoupling of the PI3K pathway via mutation necessitates combinatorial treatment in HER2+ breast cancer. PLoS One.

[bib21] Korkut A., Wang W., Demir E., Aksoy B.A., Jing X., Molinelli E.J., Babur Ö., Bemis D.L., Onur Sumer S., Solit D.B. (2015). Perturbation biology nominates upstream–downstream drug combinations in RAF inhibitor resistant melanoma cells. Elife.

[bib22] Kuhn M., Wickham H. (2020). Tidymodels: a collection of packages for modeling and machine learning using tidyverse principles. https://www.tidymodels.org.

[bib23] Long G.V., Stroyakovskiy D., Gogas H., Levchenko E., de Braud F., Larkin J., Garbe C., Jouary T., Hauschild A., Grob J.J. (2014). Combined BRAF and MEK inhibition versus BRAF inhibition alone in melanoma. N. Engl. J. Med..

[bib24] Martin T.D., Cook D.R., Choi M.Y., Li M.Z., Haigis K.M., Elledge S.J. (2017). A role for mitochondrial translation in promotion of viability in K-ras mutant cells. Cell Rep..

[bib25] Nowak-sliwinska P., Weiss A., Ding X., Dyson P.J., van den Bergh H., Griffioen A.W., Ho C.m. (2016). Optimization of drug combinations using feedback system control. Nat. Protoc..

[bib26] Nyman E., Stein R.R., Jing X., Wang W., Marks B., Zervantonakis I.K., Korkut A., Gauthier N.P., Sander C. (2020). Perturbation biology links temporal protein changes to drug responses in a melanoma cell line. PLoS Comput. Biol..

[bib27] Ozkan-Dagliyan I., Diehl J.N., George S.D., Schaefer A., Papke B., Klotz-Noack K., Waters A.M., Goodwin C.M., Gautam P., Pierobon M. (2020). Low-dose vertical inhibition of the RAF-MEK-ERK cascade causes apoptotic death of KRAS mutant cancers. Cell Rep..

[bib28] Core Team R. (2021). https://www.R-project.org/.

[bib29] Ryan M.B., Fece de la Cruz F., Phat S., Myers D.T., Wong E., Shahzade H.A., Hong C.B., Corcoran R.B. (2019). Vertical Pathway Inhibition Overcomes Adaptive Feedback Resistance to KRASG12C Inhibition. Clin. Cancer. Res..

[bib30] Saez-Rodriguez J., Alexopoulos L.G., Epperlein J., Samaga R., Lauffenburger D.A., Klamt S., Sorger P.K. (2009). Discrete logic modelling as a means to link protein signalling networks with functional analysis of mammalian signal transduction. Mol. Syst. Biol..

[bib31] Saez-Rodriguez J., Alexopoulos L.G., Zhang M., Morris M.K., Lauffenburger D.A., Sorger P.K. (2011). Comparing signaling networks between normal and transformed hepatocytes using discrete logical models. Cancer Res..

[bib32] Soule H.D., Maloney T.M., Wolman S.R., Peterson W.D., Brenz R., McGrath C.M., Russo J., Pauley R.J., Jones R.F., Brooks S.C. (1990). Isolation and characterization of a spontaneously immortalized human breast epithelial cell line, MCF-10. Cancer Res..

[bib33] Torrance C.J., Agrawal V., Vogelstein B., Kinzler K.W. (2001). Use of isogenic human cancer cells for high-throughput screening and drug discovery. Nat. Biotechnol..

[bib34] van Buggenum J.A.G., Gerlach J.P., Tanis S.E.J., Hogeweg M., Jansen P.W.T.C., Middelwijk J., van der Steen R., Vermeulen M., Stunnenberg H.G., Albers C.A., Mulder K.W. (2018). Immuno-detection by sequencing enables large-scale high-dimensional phenotyping in cells. Nat. Commun..

[bib35] Van Cutsem E., Cuyle P.J., Huijberts S., Yaeger R., Schellens J.H.M., Elez E., Tabernero J., Fakih M., Montagut C., Peeters M. (2018). BEACON CRC study safety lead-in (SLI) in patients with BRAFV600E metastatic colorectal cancer (mCRC): efficacy and tumor markers. J. Clin. Oncol..

[bib36] Van Cutsem E., Huijberts S., Grothey A., Yaeger R., Cuyle P.J., Elez E., Fakih M., Montagut C., Peeters M., Yoshino T. (2019). Binimetinib, encorafenib, and cetuximab triplet therapy for patients with *BRAF* V600e–mutant metastatic colorectal cancer: safety lead-in results from the phase III BEACON colorectal cancer study. J. Clin. Oncol..

[bib37] Veninga V., Voest E.E. (2021). Tumor organoids: opportunities and challenges to guide precision medicine. Cancer Cell.

[bib38] Virtanen P., Gommers R., Oliphant T.E., Haberland M., Reddy T., Cournapeau D., Burovski E., Peterson P., Weckesser W., Bright J., SciPy 1.0 Contributors (2020). SciPy 1.0: fundamental algorithms for scientific computing in Python. Nat. Methods.

[bib39] Vis D.J., Bombardelli L., Lightfoot H., Iorio F., Garnett M.J., Wessels L.F. (2016). Multilevel models improve precision and speed of IC50 estimates. Pharmacogenomics.

[bib40] Weinstein I.B. (2002). Addiction to oncogenes–the achilles heal of cancer. Science.

[bib41] Weiss A., Berndsen R.H., Ding X., Ho C.M., Dyson P.J., van den Bergh H., Griffioen A.W., Nowak-Sliwinska P. (2015). A streamlined search technology for identification of synergistic drug combinations. Sci. Rep..

[bib42] Wolfram Research, Inc. (2019).

[bib43] Wu X., Renuse S., Sahasrabuddhe N.A., Zahari M.S., Chaerkady R., Kim M.S., Nirujogi R.S., Mohseni M., Kumar P., Raju R. (2014). Activation of diverse signalling pathways by oncogenic PIK3CA mutations. Nat. Commun..

[bib44] Zhao W., Li J., Chen M.J.M., Luo Y., Ju Z., Nesser N.K., Johnson-Camacho K., Boniface C.T., Lawrence Y., Pande N.T. (2020). Large-Scale characterization of drug responses of clinically relevant proteins in cancer cell lines. Cancer Cell.

[bib45] Zhong L., Li Y., Xiong L., Wang W., Wu M., Yuan T., Yang W., Tian C., Miao Z., Wang T., Yang S. (2021). Small molecules in targeted cancer therapy: advances, challenges, and future perspectives. Signal Transduct. Targeted Ther..

[bib46] Zoetemelk M., Ramzy G.M., Rausch M., Koessler T., van Beijnum J.R., Weiss A., Mieville V., Piersma S.R., de Haas R.R., Delucinge-Vivier C. (2020). Optimized low-dose combinatorial drug treatment boosts selectivity and efficacy of colorectal carcinoma treatment. Mol. Oncol..

