# Supplementary material for: Identifying mutant-specific multi-drug combinations using comparative network reconstruction
Source: iScience. 2022 Jul 15;25(8):104760. doi: 10.1016/j.isci.2022.104760 (PMC9385552; doi:10.1016/j.isci.2022.104760)
Supplement: Document S1. Figures S1–S6 [file mmc1.pdf]

iScience, Volume *25*

Supplemental information

Identifying mutant-specific multi-drug combinations using comparative network reconstruction

Evert Bosdriesz, João M. Fernandes Neto, Anja Sieber, René Bernards, Nils Blüthgen, and Lodewyk F.A. Wessels

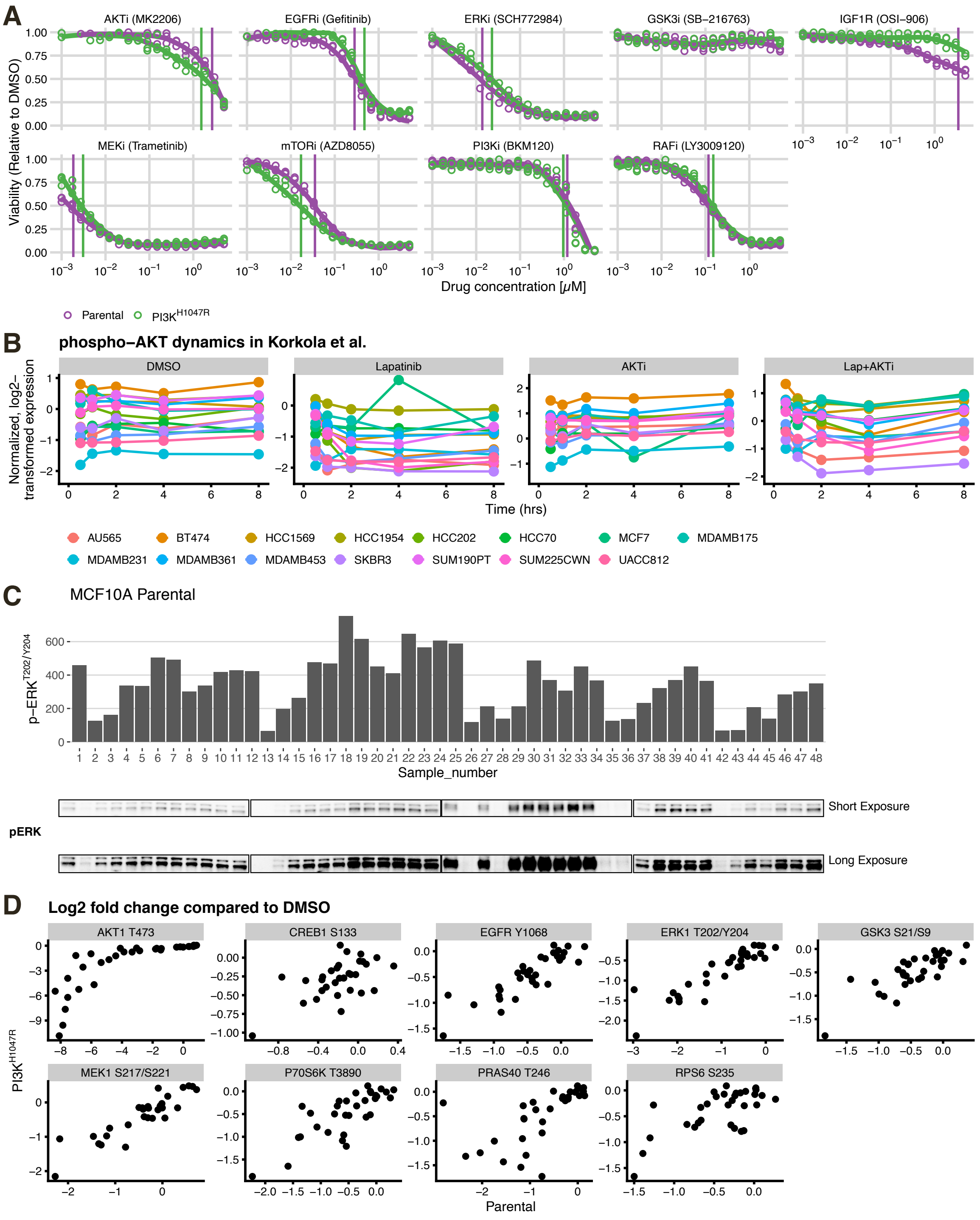

Figure S1: **Profiling signaling and viability response of MCF10A parental en PI3K^{H1047R} cells to drug perturbations. Related to Figure 2. A.** Dose-response curves of the inhibitors used in this study. **B.** Dynamics of AKT activity after PI3K pathway inhibition from Korkola *et al.* (2015). **C.** Correlation between phospho-ERK quantification using Luminex (top) and Western blot (bottom). **D.** Correlation between the response in parental (x-axis) and PI3K^{H1047R} (y-axis) cells. Response is defined as log$_2$-fold change compared to DMSO controls.

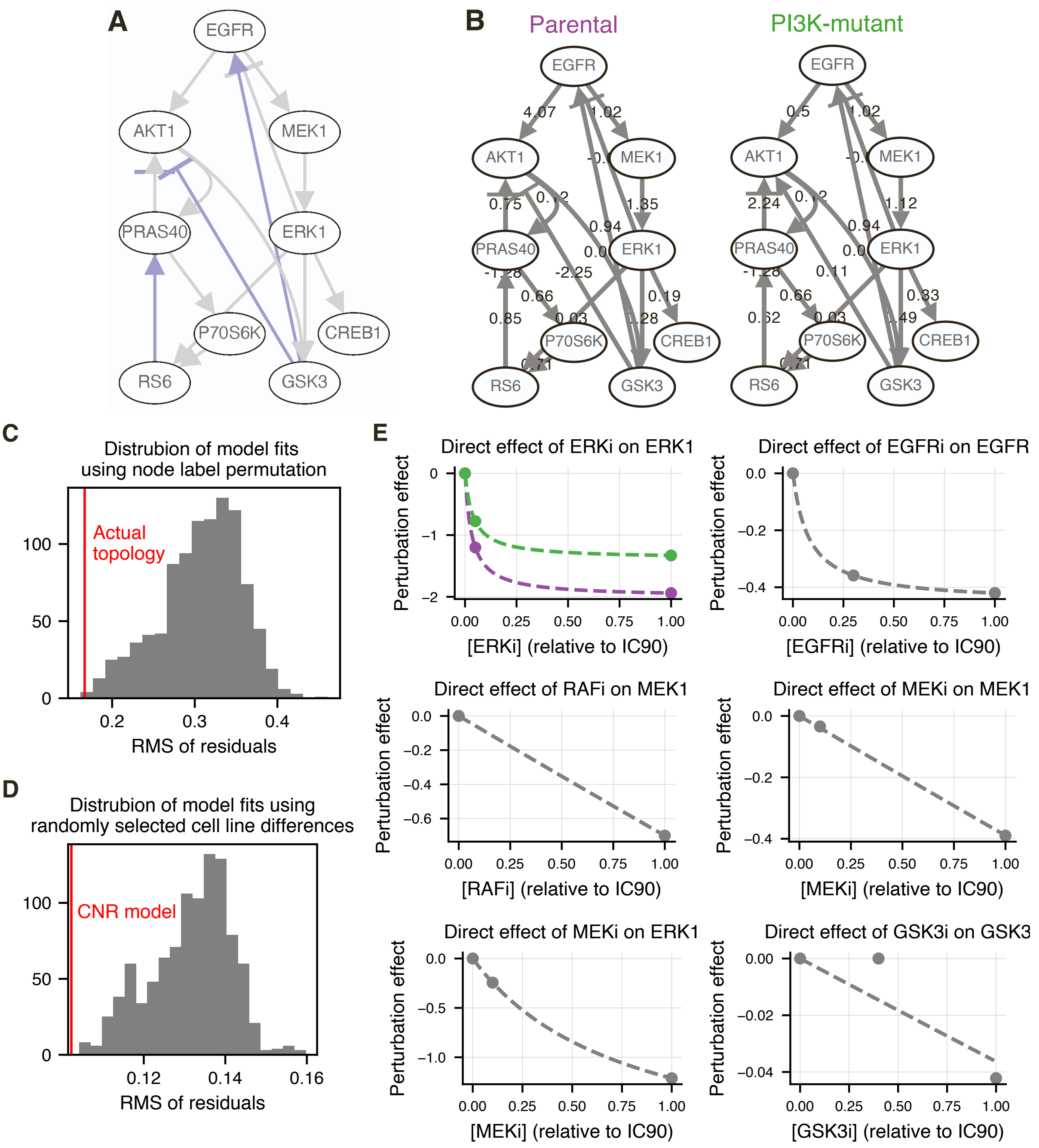

Figure S2: **Comparative Network Reconstruction of MCF10A Parental and PI3K^{H1047R} cells. Related to Figure 3. A.** Network topology used for modeling. Edges used as prior information are indicated in gray. Edges added in a leave one out cross validation loop are indicated in purple. **B.** Network models of the parental (left) and PI3K^{H1047R} cells. Edge labels indicate reconstructed interaction strengths (r_{ij} terms in Equation 2 and 3). **C.** Significance of the network topology used. Distribution of hte residual of 1000 models which were randomize by permuting the node labels (while keeping inhibitor-target node relations correct). Only one of the random models had RMS of residuals lower than the model we used ($p = 0.001$). **D.** Significance of identified differences between the cell lines. Distribution of the residuals of 1000 model optimizations in which random edges were selected to allow to differ between the two cell. The selected model has a better model fit than all 1000 of these models ($p < 0.001$). **E.** The estimated direct effect of different inhibitors on their target, as a function of applied inhibitor concentration (s_{ij} terms in Equations 2 and 3). Points indicate the estimated effects obtained from the CNR reconstruction, at the concentrations used in the perturbation experiments. The dashed lines indicate the interpolated curves between these points. (c.f. Materials and Methods, Equation 5)

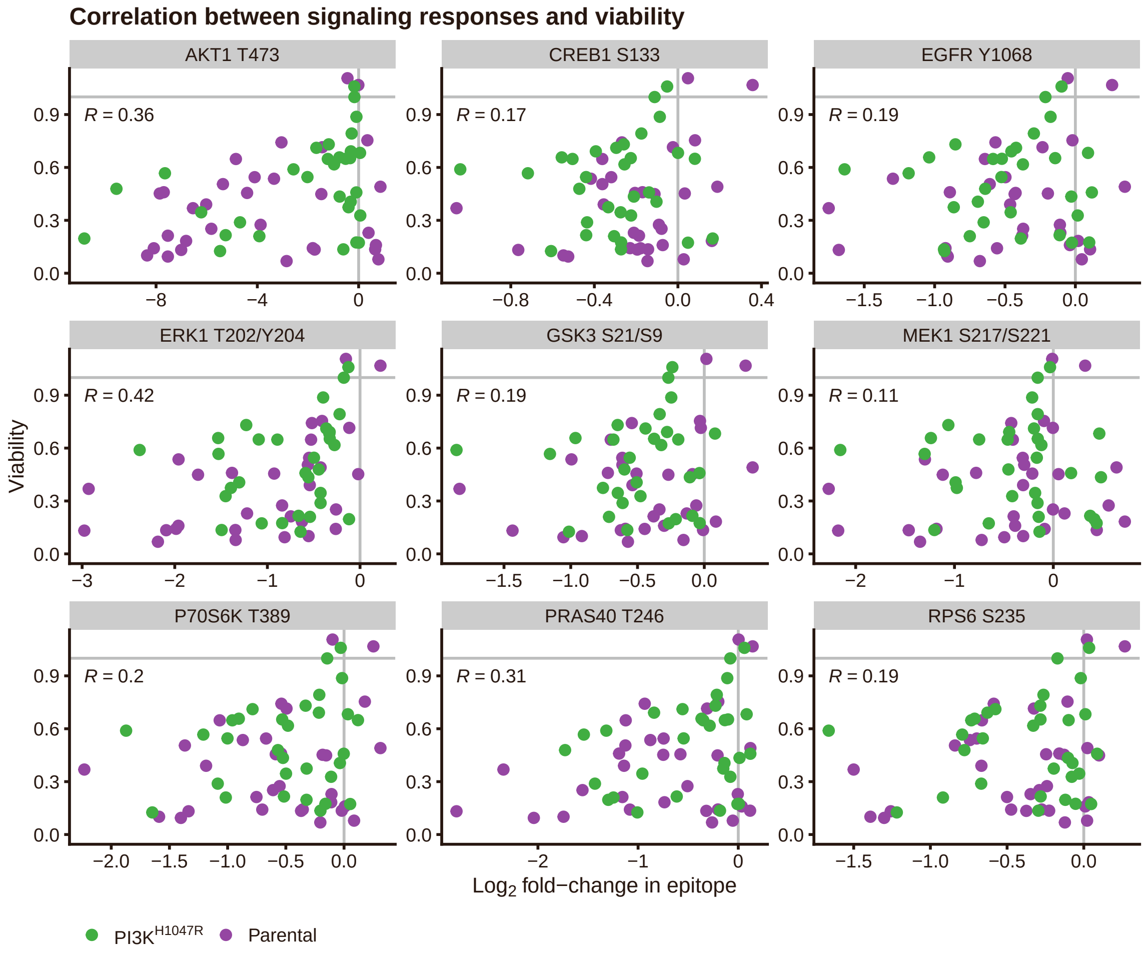

Figure S3: **Correlation between node response and cell viability of all measured nodes. Related to Figure 4.** *R* indicates Pearson correlation. Log_2 fold-changes and viabilities are all relative to DMSO control.

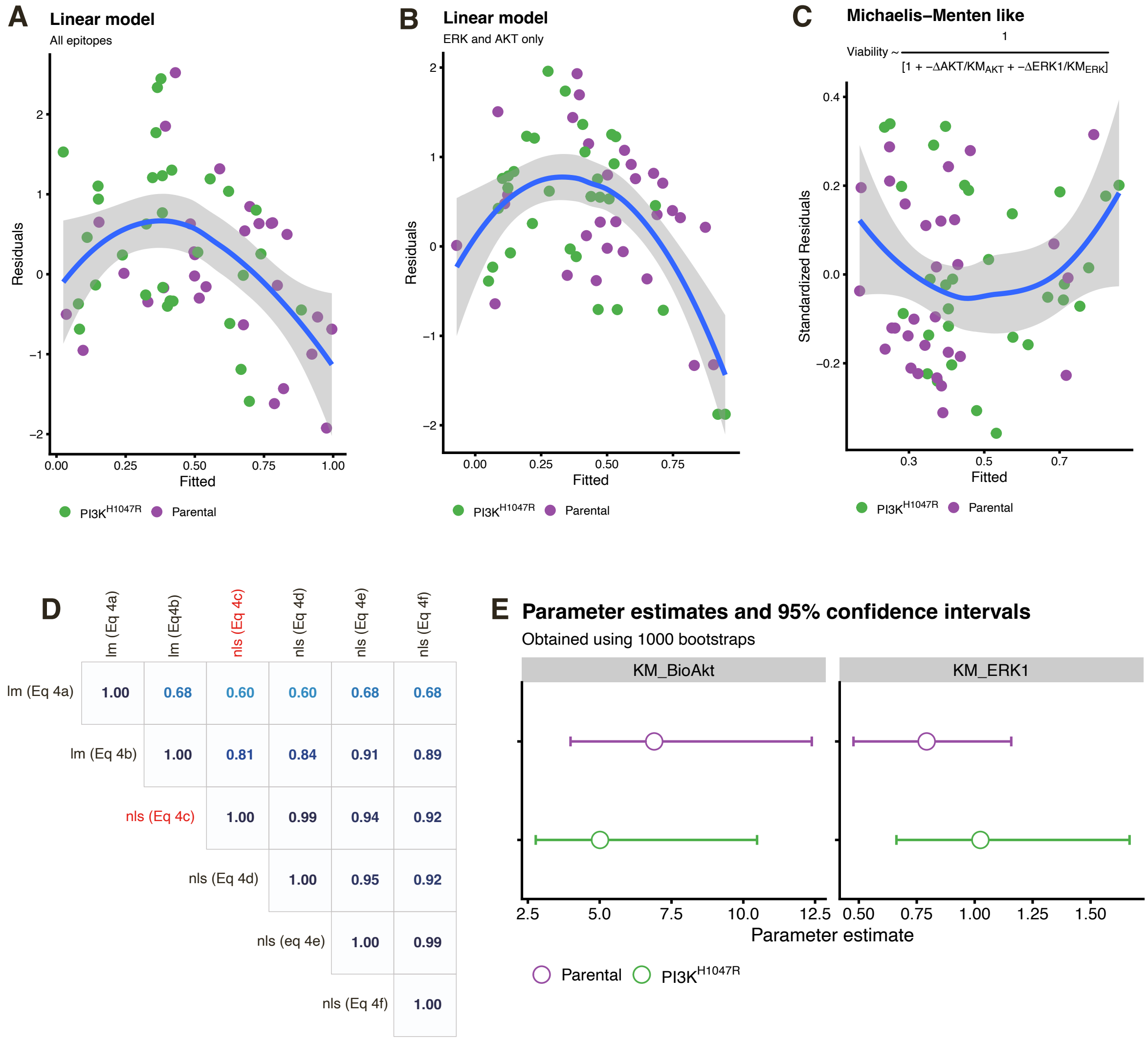

Figure S4: **Evaluation of model fits relating signaling response to cell viability. Related to Figure 4. A-C.** Residuals as a function of fitted values for the model fits. x-axis is capped at 1.0 because we are only interested in to predict inhibition of cell viability. **A.** Linear model with all epitopes as predictor (Equation 4a). **B.** Linear model with only R_{AKT} and R_{ERK} as predictor (Equation 4b). **C.** Non-linear model that gave the best fit (Equation 4c). **D.** Pearson correlation between model predictions of all models tested show that the predictions of all non-linear models are highly similar. Predictions are obtained from the leave-one-out cross-validation. The selected model (Equation 4c) is highlighted in red. The plot is generated using the `corrplot`-function of the corrplot R-package (Wei and Simko, 2021). **E.** Bootstrapping intervals of the estimated values for the parameters $K_{M,AKT}$ and $K_{M,ERK}$ in Equation 4c

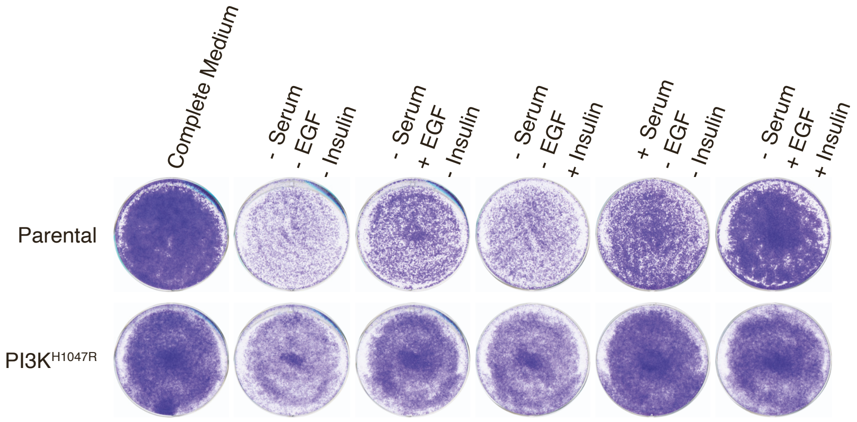

Figure S5: **Growth of MCF10A parental and PI3K^{H1047R} cells in different growth media. Related to the section "Prediction and validation of Selective multi-drug combinations".** In contrast to the parental cells the PI3K mutant cells grow well in the absence of serum if either Insulin or EGF is provided.

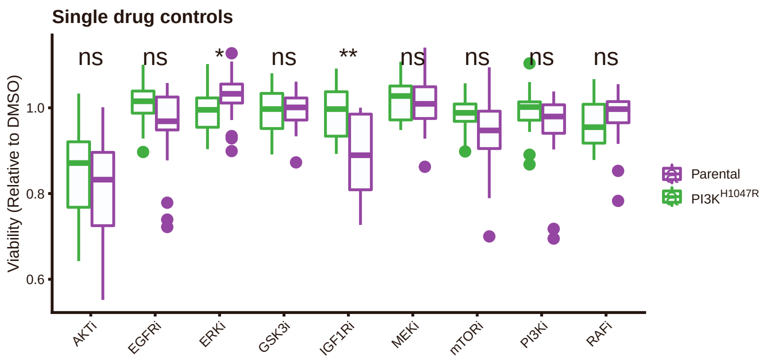

Figure S6: **Mono-drug treatments show little to no (anti)-selectivity. Related to Figure 5.** Viability of the low-dose single-drug controls, all measured at their IC_{10}. Except for IGF1Ri, none of the drugs show selectivity towards the parental cells. Treatments were performed in 8 replicates.
